# Treatment with crocin attenuates cardiac metabolic disturbances and subsequent inflammation in streptozotocin-induced diabetes

**DOI:** 10.1007/s11010-025-05320-0

**Published:** 2025-06-03

**Authors:** Dimitra Palioura, Konstantinos Feidantsis, Antigone Lazou

**Affiliations:** 1 Laboratory of Animal Physiology, School of Biology, Aristotle University of Thessaloniki, 54124 Thessaloniki, Greece; 2 Department of Fisheries and Aquaculture, University of Patras, 26504 Mesolonghi, Greece; 3 School of Life and Health Sciences, University of Nicosia, CY-1700 Nicosia, Cyprus

**Keywords:** Crocin, Diabetes mellitus, Diabetic cardiomyopathy, Myocardial metabolism, Glucose metabolism, Lipid metabolism

## Abstract

Diabetes mellitus (DM) is a metabolic disorder closely associated with cardiac dysfunction. Natural products are considered potential candidates for the management of DM. Crocin, a natural carotenoid derived from saffron, has been reported to possess several pharmacological properties, including cardioprotective effects. The aim of the present study was to investigate the beneficial effects of crocin on the metabolic derangement of the diabetic myocardium. Streptozotocin (STZ)-induced diabetic rats were treated with 10 mg/kg of crocin daily for two weeks. Oral administration of crocin normalized blood glucose, HbA1c, triglycerides, total cholesterol, LDL, and HDL levels. Notably, crocin reduced the elevated protein levels of cardiac PPARα and PPARδ—major transcriptional regulators of cardiac metabolism—back to normal. Consequently, the expression of the fatty acid (FA) transporter CD36 was downregulated, the activity of the FA oxidation enzyme 3-hydroxyacyl-CoA dehydrogenase (HOAD) was decreased, and intramyocardial triglyceride accumulation returned to physiological levels. Furthermore, crocin improved glucose uptake and metabolism in the diabetic myocardium, as evidenced by increased Akt phosphorylation, translocation of GLUT4 to the plasma membrane, enhanced activity of pyruvate kinase, and downregulation of pyruvate dehydrogenase kinase 4 (PDK4). Importantly, the stimulatory effect of crocin on Akt phosphorylation was also confirmed in isolated cardiac myocytes exposed to high glucose, further supporting its direct role in modulating glucose signaling pathways. Crocin treatment also reduced STZ-induced elevations in the levels of inflammatory cytokines—interleukin-6 (IL-6) and tumor necrosis factor-α (TNF-*α*)—as well as the phosphorylation of IκBα, bringing them close to basal levels. Overall, these findings suggest that crocin activates Akt signaling and thereby alleviates diabetes-induced metabolic disturbances by restoring the balance between glucose and fatty acid utilization in the hearts of STZ-induced diabetic rats. Therefore, crocin supplementation may represent a promising approach for the development of natural compound-based adjunct therapies for diabetic cardiomyopathy.

## Introduction

Diabetes mellitus (DM) is a chronic metabolic disorder characterized by sustained hyperglycaemia and hyperlipidaemia [1]. Among its many complications, diabetic cardiomyopathy (DCM) prevails as a leading cause of morbidity and mortality in diabetic patients [2, 3].

DCM is marked by structural and functional alterations in the heart, including myocardial fibrosis, hypertrophy, and impaired contractility, which ultimately lead to heart failure [4]. A key driver of DCM is the dysregulation of cardiac metabolism, where the heart shifts from its primary reliance on fatty acid oxidation to increased glucose utilization, resulting in mitochondrial dysfunction, impaired energy production, and accumulation of toxic lipid intermediates [5, 6]. These metabolic disturbances not only compromise cardiac efficiency but also trigger oxidative stress and inflammation, further exacerbating cardiac damage and dysfunction [4]. However, despite advances in understanding the pathogenesis of DCM, effective therapeutic strategies to mitigate these metabolic and inflammatory changes remain limited [4].

Over the past years, there has been growing interest in the potential therapeutic use of plant derived natural products, defined as nutraceuticals [7–9]. Crocin, the main active constituent of saffron (*Crocus sativus* L), is a water-soluble natural carotenoid with many beneficial pharmacological properties, reviewed in [10, 11]. Crocin can ameliorate oxidative stress and inflammation in various disease models, including cardiovascular disorders [12–14] and can also have antihypertensive and direct anti-atherogenic actions [13, 15]. In addition, increasing preclinical data have highlighted the glucose and lipid lowering effect of crocin making it a potential candidate for addressing metabolic disturbances in diabetic conditions [12, 13, 16–18]. Our own data have also demonstrated that treatment with crocin attenuates cardiac dysfunction and remodeling in STZ-induced diabetic rats by ameliorating cardiac hypertrophy and fibrosis, inhibiting excessive myocardial apoptosis and normalizing autophagy [19].

Despite these promising findings, the specific effects of crocin on cardiac metabolic disturbances in the context of diabetes remain underexplored. Given the central role of metabolic dysregulation in the development of cardiac dysfunction and the progression of diabetic cardiomyopathy [20], in the present study, we aimed to determine whether crocin ameliorates the metabolic derangement of the diabetic myocardium. To this end, we sought to investigate the effect of treatment with crocin on enzymes and other regulatory factors involved in both glucose and lipid metabolism in the heart, as well as on cardiac inflammatory markers in an STZ-induced diabetes animal model.

## Materials and methods

### Animal care and treatment

Animals received proper care in compliance with the ‘‘Principles of laboratory animal care’’ published by the Greek Government (2013/56) based on EU regulations (2010/63EE). The animal study was reviewed and approved by the Committee on the Ethics of Animal Experiments of the Directorate of Veterinary Services of Prefecture of Thessaloniki (No 399301/2966). Male Wistar rats (200 g body weight) were housed under standard conditions with a constant 12:12 h light/dark cycle and temperature, fed a standard diet and had access to tap water ad libitum. Rats were randomized into three groups (*n* = 4 for each group): (a) control group, treated with citrate buffer vehicle, (b) diabetic animals, treated with STZ and (c) diabetic animals treated with 10 mg/kg body weight crocin. The concentration of crocin (PubChem CID: 16211383) (Sigma-Aldrich) used in the present study were according to Feidantsis et al [19]. Diabetes mellitus was induced with a single dose of STZ (65 mg/kg dissolved in citrate buffer at pH 4.5). Crocin was administered orally (dissolved in drinking water), daily for two weeks after 6 weeks of diabetes induction. Before any treatment with crocin, and to ensure that all animals were diabetic, blood was collected from the lateral tail vein and glucose levels were determined. Animals with fasting blood glucose levels exceeding 200 mg/dl were considered diabetic. All efforts were made to minimize suffering. Specifically, rats were anesthetized by isoflurane. Heart tissue was isolated and immediately frozen in liquid nitrogen and stored at – 80 ℃ until further analysis.

### Determination of blood biochemical profile

Glucose, total cholesterol, triglycerides, HDL cholesterol and LDL cholesterol levels were determined in plasma, and HbA1c levels in whole blood samples, based on enzymatic–colorimetric assays using commercial kits (Spinreact, Spain, glucose kit, cod. 1001191; total cholesterol kit, cod. MD41021; triglycerides kit, cod. 1001312; HDL cholesterol kit, cod. MD1001096; LDL cholesterol kit, cod. SP41023; HbA1c kit, cod. MD43100).

### Isolation of adult rat cardiac myocytes and treatments

Cardiac myocytes preparations used for the experiments were obtained from different animal groups (*n* = 4). Ventricular myocytes were isolated by cardiac retrograde aortic perfusion and collagenase treatment in a Langendorff apparatus as described previously [21]. Briefly, the hearts were perfused with warm Krebs-Henseleit (KH) solution (25 mM NaHCO_3_, 4.7 mM KCl, 118.5 mM NaCl, 1.2 mM MgSO4·7H_2_O, 1.2 mM KH_2_PO4, 10 mM glucose, pH 7.4) supplemented with 10 mM butanedione monoxime and 5*μ*M CaCl_2_, for 5 min. Then, the heart was perfused with KH solution supplemented with 1 mg/ml BSA, 10 mM butanedione monoxime, 50*μ*M CaCl_2_ and 180U/ml collagenase type II (digestion buffer). After digestion, the softened heart was mechanically dissociated and passed through a nylon mesh. The cells suspension was washed twice with KH solution with 50*μ*M CaCl_2_ and 1 mg/ml BSA. Eventually, isolated cardiomyocytes were resuspended in KH solution with 50*μ*M CaCl_2_, which was gradually increased up to final 1 mM CaCl_2_. Preparations were considered satisfactory only if the yield of rod-shaped cells was more than 70%. Experiments were performed 30 min after dissociation. To mimic the effect of high glucose in the blood plasma, cardiac myocytes were exposed to glucose, at a final concentration of 25 mmol/l. Crocin was added to high glucose medium at a concentration of 1 *μ*mol/l or 10 *μ*mol/l for 3 hours. The concentrations of crocin were chosen according to preliminary experiments and the existing literature [19, 22, 23].

### Isolation of RNA and quantitative PCR (qPCR)

Total RNA was extracted from frozen tissue samples using NucleoZol (Macherey-Nagel, Duren, Germany) according to the manufacturer’s instructions. The RNA was resuspended in 0.1% (v/v) diethylpyrocarbamate (DEPC)-treated water and its concentration was determined by absorbance at 260 nm. The integrity of isolated RNA was verified on a 1% agarose gel. cDNA synthesis of 1 μg of total RNA was performed using PrimeScript RT reagent kit (#RR037 A, Takara, Kusatsu, Japan). Reaction, in a total volume of 20 *μ*l, was carried out for 15 min at 37 °C, and was terminated by heat inactivation of the reverse transcriptase at 85 °C for 5 sec. qPCR analysis was performed using a Real-Time PCR System (Applied Biosystems) with a specific set of primers for each gene. The sequences of the sense and antisense primers used for amplification were: *pdk4*: 5’-AGCTGCTGGACTTCGGTTCA-3’ and 5’-CGTTCAGGGAGGATGTCAA-3’; *cd36*: 5’-TGTGTTTGGAGGCATTCTCA-3’ and 5’-TGGGTTTTGCACATCAAAGA-3’; *β-actin*: 5’-GCCCTGAGGCACTCTTCCA-3’ and 5’- CGGATGTCCACGTCACACTTC-3’. Each reaction mix contained 5 *μ*l KAPA SYBR FAST qPCR Master Mix (KAPA Biosystems, Wilmington, MA, USA), 0.3 *μ*l oligonucleotides (10 pmol each of forward and reverse primers) and 2 *μ*l cDNA. qPCR analysis of actin was performed as endogenous control. PCR conditions were 95 °C for 20 s, followed by 40 cycles of 95 °C for 3 s and 60 °C for 30 s. Following qPCR, dissociation curve analysis was routinely performed to check for aberrant amplification products (e.g., primers-dimers). Gene expression was normalized to *β-actin*. Relative changes in expression were calculated using the 2^−(−ΔΔct)^ method.

### Subcellular fractionation

For the separation of membranous and cytosolic fractions, cardiac tissue samples were lysed in a buffer containing 12.5 mM Tris-HCl, pH 7.4, 2.5 mM EGTA, 1 mM EDTA, 100 mM NaF, 5 mM dithiothreitol, 300 mM phenylmethylsulfonyl fluoride, 120 μΜ pepstatin A, 200 μΜ leupeptin, 10 μΜ trans-epoxy-succinyl-L- leucyl-amido (4-guanidino) butane and 0.05% digitonin. The extracts were incubated for 5 min at 4 °C and then they were centrifuged at 12000*g* for 15 min, at 4 °C. The supernatant containing the cytosolic fraction was transferred to another tube on ice. The remaining pellet was washed twice in 100μl of lysis buffer, centrifuged and finally resuspended in the same volume of lysis buffer containing 1% (v/v) Triton-X. These samples were lightly vortexed, left on ice for 20 min and then they were centrifuged at 12000*g* for 15 min, at 4 °C. The supernatant containing the membranous fractions were collected. Total protein, in cytosolic and membranous extracts, was measured using the Bradford assay (BioRad).

### Preparation of heart tissue and cell protein extracts and immunoblotting

To determine the protein levels of PPARα, PPARβ/δ, GLUT-4, IL-6, and TNFα, as well as the phosphorylation levels of Akt and ΙκΒα, standard SDS-PAGE and immunoblotting protocols were used. Frozen whole heart samples and cell suspensions were lysed on ice in a buffer containing 20 mM β-glycerophosphate, 20 mM HEPES pH 7.5, 20 mM NaF, 2 mM EDTA, 0.2 mM Na_3_VO_4_,10 mM benzamidine, 5 mM DTT, 0.3 mM PMSF, 0.2 mM leupeptin, 0.01 mM E64 and 1% (v/v) Triton X-100. Lysates were incubated on ice for 10 min and then centrifuged at 10,000*g* for 15 min at 4 °C. Protein extracts were collected, and total protein was measured using the Bradford assay (BioRad). Equal amounts of protein were separated on 10% (w/v) acrylamide and 0.275% (w/v) bis-acrylamide gels and transferred onto 0.45 μm nitrocellulose membranes (Schleicher and Schuell, Keene, USA). Subsequently, the membranes were incubated overnight with the appropriate primary antibodies: anti-PPARδ (#101720, Cayman), anti-PPARα (#101710, Cayman), anti-phospho Akt (#9271, Cell Signaling), anti-Akt (#4691, Cell Signaling), anti-Glut-4 (#2213, Cell Signaling), anti-Na-K ATPase (#3010, Cell Signaling), anti-IL-6 (#CSB-PA06757 A0Rb, Cusabio), anti-TNFα (#CSB-PA07427 A0Rb, Cusabio), anti-α-tubulin (#2125, Cell Signaling) and anti-β-actin (#3700, Cell Signaling). After washing in TBST (3x5 min) the blots were incubated with horseradish peroxidase-linked secondary antibodies, washed again in TBST (3x5 min), and the bands were detected using enhanced chemiluminescence (Cell Signaling, Boston, USA) with exposure to Fuji Medical X-ray films. Bands were quantified by laser-scanning densitometry.

### Determination of cardiac enzymes activity

Enzyme activities of 3-hydroxyacyl CoA dehydrogenase (HOAD; E.C. 1.1.1.35) and pyruvate kinase (PK; E.C. 2.7.1.40) were determined spectrophotometrically following the oxidation of NADH at 340 nm (mM extinction coefficient = 6.22) in heart tissue homogenates as described in detail in [24, 25]. Heart tissue samples were homogenized in imidazole buffer containing 50 mM imidazole, 1 mM EDTA, 2 mM MgCl2, pH 7.6. Homogenates were centrifuged at 7000 g for 5 min at 4 °C. PK enzymatic activity was determined in the presence of 50 mM imidazole buffer pH 7.0, 50 mM KCl, 2 mM MgCl2, 0.15 mM NADH, 2 mM ADP, 2 mM 2-phosphoenolpyruvate and 0.005% lactate dehydrogenase, while HOAD activity was determined in the presence of 50 mM imidazole buffer pH 7, 1 mM EDTA, 0.15 mM NADH, 1 mM KCN and 0.1 mM acetoacetyl-CoA. Enzyme activities are expressed as micromoles of substrate min/mg protein.

### Determination of heart triglyceride content

Triglyceride content in the heart was determined according to the method of Babu et al. [26]. Snap frozen heart tissue samples were homogenized in a 50 mM Tris buffer pH 8.5 containing 5 mM EDTA and 30 mM mannitol using a pestle. Later, 0.25% v/v 10M KOH and a mixture of chloroform/methanol (2:1) were added to aid the extraction of lipids. Homogenates were centrifuged at 13,000 g twice and the top aqueous phase was discarded. The bottom layer containing the triglycerides was left to evaporate overnight at room temperature and the remaining pellet was resuspended in a butanol mixture (60% butanol, 33.3% Triton-X100, 6.6% methanol). Triglycerides content was eventually determined spectrophotometrically using the SpinReact Triglycerides-LQ Kit #41030.

### Statistical analysis

Data are presented as mean ± SEM. Statistical analyses (ANOVA followed by Student-Newman-Keuls multiple comparisons test) were performed using GraphPad Instat (GraphPad Software, San Diego, CA, USA) and significance was established at p< 0.05.

## Results

### Treatment with crocin alleviates the diabetic-impaired biochemical profile

**Fig. 1:**
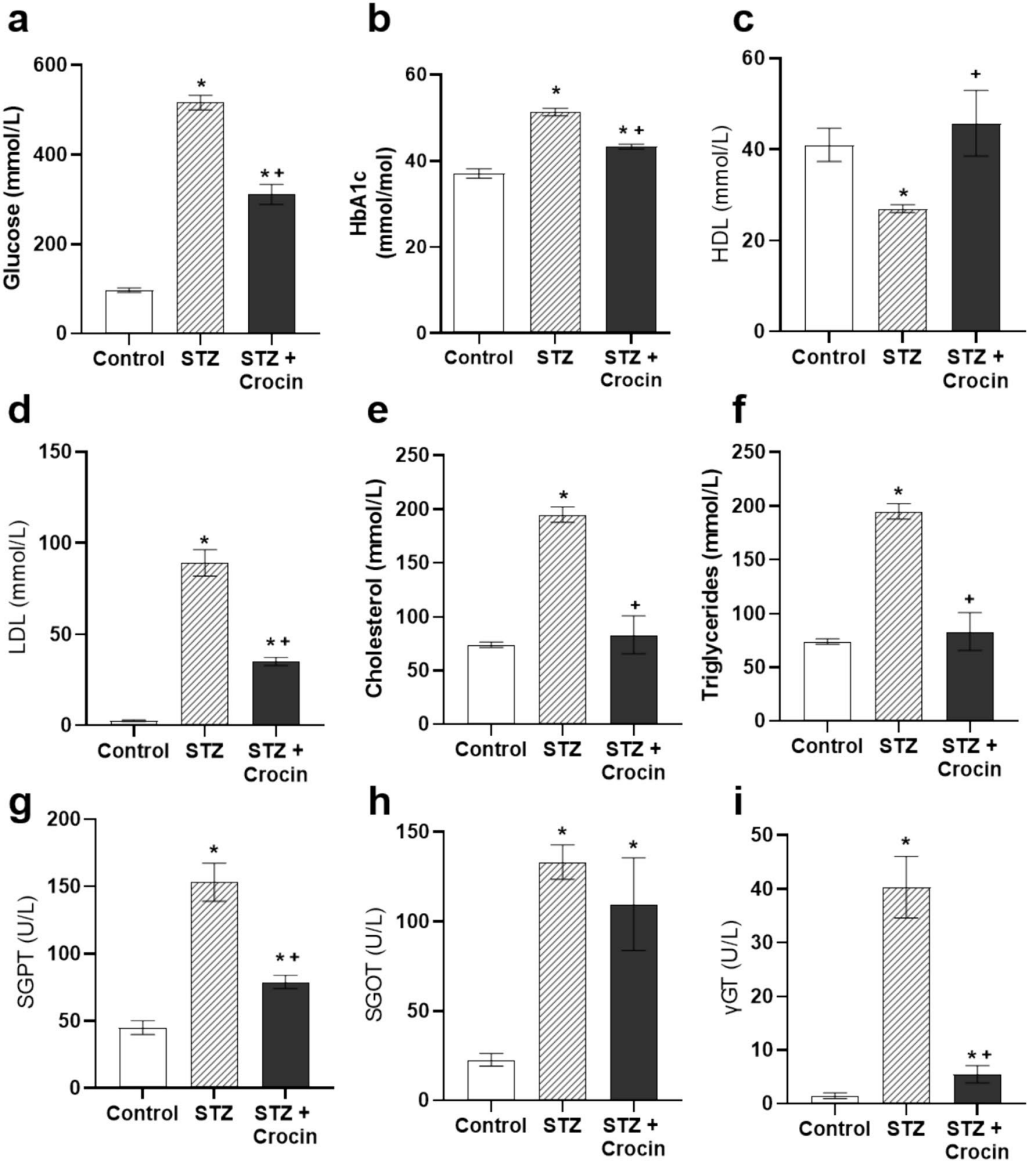
Treatment with crocin normalizes the disturbed plasma biochemical profile in STZ-diabetic rats. **a** Glucose, **b** HbA1c, **c** HDL, **d** LDL, **e** total cholesterol, **f** triglycerides, **g** SGPT, **h** SGOT, **i** γGT levels in the blood plasma of control, STZ - treated and STZ + crocin - treated rats. Data are presented as mean ± SEM; *p < 0.05 compared with control, +*p* < 0.05 compared with STZ-treated group; one-way ANOVA followed by Student-Newman-Keuls multiple comparisons test; *n* = 4

Hyperglycemia and hyperlipidemia in diabetes are reflected in abnormal plasma metabolites levels. Oral consumption of crocin led to a reduction in plasma glucose (Figure 1a) and HbA1c levels (Figure 1b), both of which were significantly elevated in the diabetic group. Moreover, crocin treatment normalized lipid profile abnormalities in STZ-diabetic rats, including reduced HDL (Figure 1c), elevated LDL (Figure 1d), total cholesterol (Figure 1e) and triglycerides (Figure 1f). Additionally, diabetic animals exhibited increased serum levels of SGPT (Figure 1g), SGOT (Figure 1h) and γGT (Figure 1i). Treatment with crocin significantly reduced SGPT and γGT levels. Altogether, crocin treatment resulted in the normalization of plasma metabolic profile in diabetic animals.

### Treatment with crocin attenuates lipid accumulation in the diabetic heart

**Fig. 2:**
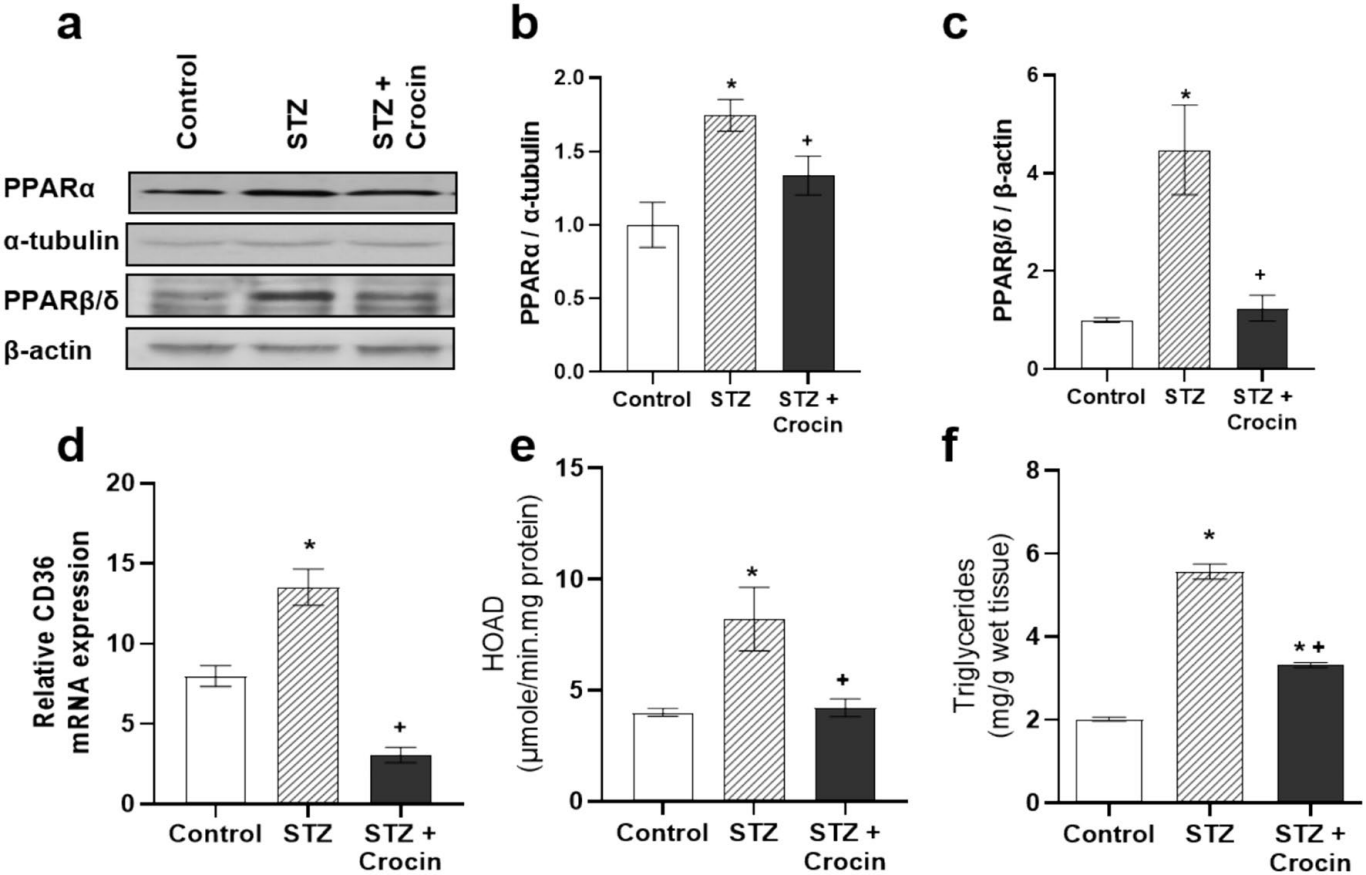
Treatment with crocin attenuates STZ-induced myocardial lipid uptake, utilization and accumulation. **a** Representative western blot images and densitometric analysis of **b** PPARα (normalized to α-tubulin) and **c** PPARδ (normalized to β-actin) protein levels in the hearts of control, STZ - treated and STZ + crocin-treated rats. **d** Relative CD36 mRNA expression (normalized to β-actin), **e** HOAD activity, **f** triglyceride content in the myocardium of control, STZ - treated and STZ + crocin - treated rats. Data are presented as mean ± SEM; *p < 0.05 compared with control group, +*p* < 0.05 compared with STZ-treated group; one-way ANOVA followed by Student-Newman-Keuls multiple comparisons test; *n* = 4

During diabetes, FAs uptake by cardiomyocytes exceeds the capacity of intracellular storage and oxidation. This leads to excessive cardiac lipid accumulation, lipotoxicity and ultimately severe cardiac dysfunction [6]. To assess the effect of crocin on the regulation of lipid metabolism in the diabetic heart, we determined the protein levels of PPARα and PPARδ, two nuclear transcriptional factors activated by FAs binding [27, 28]. Increased PPARα and PPARδ protein levels (Figure 2a–c) along with elevated mRNA expression of the FA cellular transporter, CD36 (Figure 2d) was observed in diabetic hearts, suggesting excessive FA uptake and supply. Treatment with crocin significantly reduced PPARα and PPARδ protein levels as well as CD36 expression, bringing them close to control levels in diabetic hearts treated with crocin compared to non-treated diabetic hearts (Figure 2a–d). In parallel, the enhanced activity of FAO enzyme HOAD in the diabetic hearts (Figure 2e), was restored to physiological levels in crocin-treated diabetic animals (Figure 2e). Additionally, intramyocardial triglyceride levels showed more than 2.5-fold increase in the diabetic group, but were significantly decreased when the diabetic group was treated with crocin (Figure 2f). Overall, these findings suggest that oral administration of crocin alleviates lipid accumulation and normalizes lipid utilization in the diabetic myocardium.

### Treatment with crocin enhances glucose uptake and metabolism in the diabetic heart

**Fig. 3:**
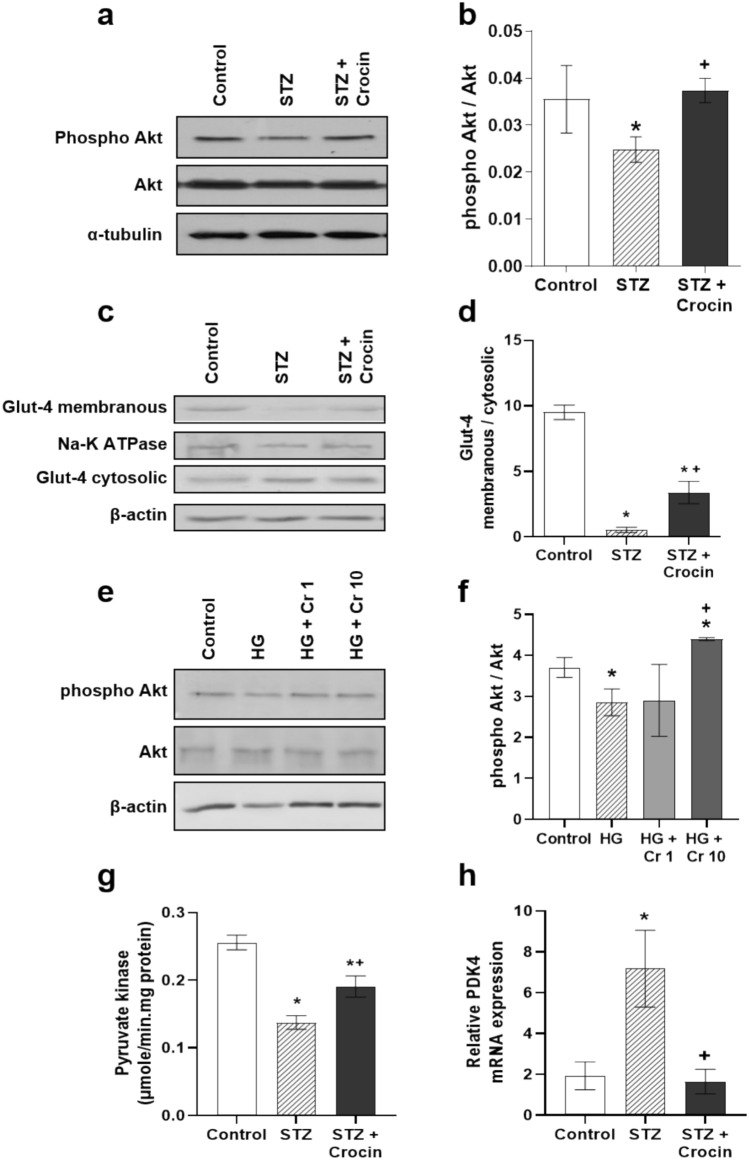
Treatment with crocin enhances glucose uptake and utilization in the diabetic myocardium. **a** Representative western blot images and **b** densitometric analysis of phosphorylated Akt (normalized to total Akt and α-tubulin). **c** Representative western blot images and **d** densitometric analysis of membranous and cytosolic GLUT-4 normalized to Na-K-ATPase and β-actin, respectively. **e** Representative western blot images and **f** densitometric analysis of phosphorylated Akt (normalized to total Akt and β-actin) in isolated adult rat cardiac myocytes treated with normal medium (Control), high glucose (HG; 25 mmol/l), HG and crocin 1 mmol/l HG + Cr 1) or HG and crocin 10 mmol/l (HG + Cr 10). **g** Pyruvate kinase activity, **h** relative PDK4 mRNA expression (normalized to β-actin) in the hearts of control, STZ - treated and STZ + crocin - treated rats. Data are presented as mean ± SEM; *p < 0.05 compared with the control group, +*p* < 0.05 compared with STZ or HG-treated group; one-way ANOVA followed by Student-Newman-Keuls multiple comparisons test; n=4

Besides lipotoxicity, poor glycemic control also contributes to severe heart complications in diabetic patients and animal models. Insulin deficiency impairs glucose uptake and glycolysis. Under normoglycemic conditions, insulin signaling in cardiomyocytes stimulates Akt-mediated translocation of glucose transporter 4 (GLUT-4) to the plasma membrane, facilitating glucose uptake. However, in the diabetic myocardium, Akt phosphorylation (Figure 3a, b) and subsequent GLUT-4 translocation to membrane were severely impaired (Figure 3c, d). Treatment with crocin significantly enhanced Akt phosphorylation (Figure 3a, b) and promoted GLUT-4 translocation (Figure 3c, d), indicating increased glucose uptake. To confirm that the effect of crocin on Akt phosphorylation in the diabetic myocardium is due to its direct action on cardiac myocytes, crocin was administrated in isolated cardiac myocytes exposed to high glucose concentrations to mimic hyperglycemia. A lower phosphorylated Akt/Akt ratio, was observed in high glucose treated cardiac myocytes compared with controls, whereas crocin treatment restored Akt phosphorylation levels, normalizing this imbalance (Fig. 3e, f).

In addition, we assessed the activity of pyruvate kinase (PK), the terminal enzyme in the glycolytic pathway, which was decreased in the diabetic hearts (Figure 3g). The stimulation of glucose metabolism in the diabetic myocardium following crocin treatment is further supported by the significant increase in PK activity, bringing it close to control levels (Figure 3e). Furthermore, diabetes-induced upregulation of PDK4 (Figure 3h), a transcriptional target of PPARα and PPARδ, was reduced after crocin treatment (Figure 3h). Overall, these findings suggest that crocin enhances myocardial glucose uptake and oxidation, restoring them to physiological levels comparable to normoglycemic animals.

### Effect of treatment with crocin on cardiac inflammation markers

**Fig. 4:**
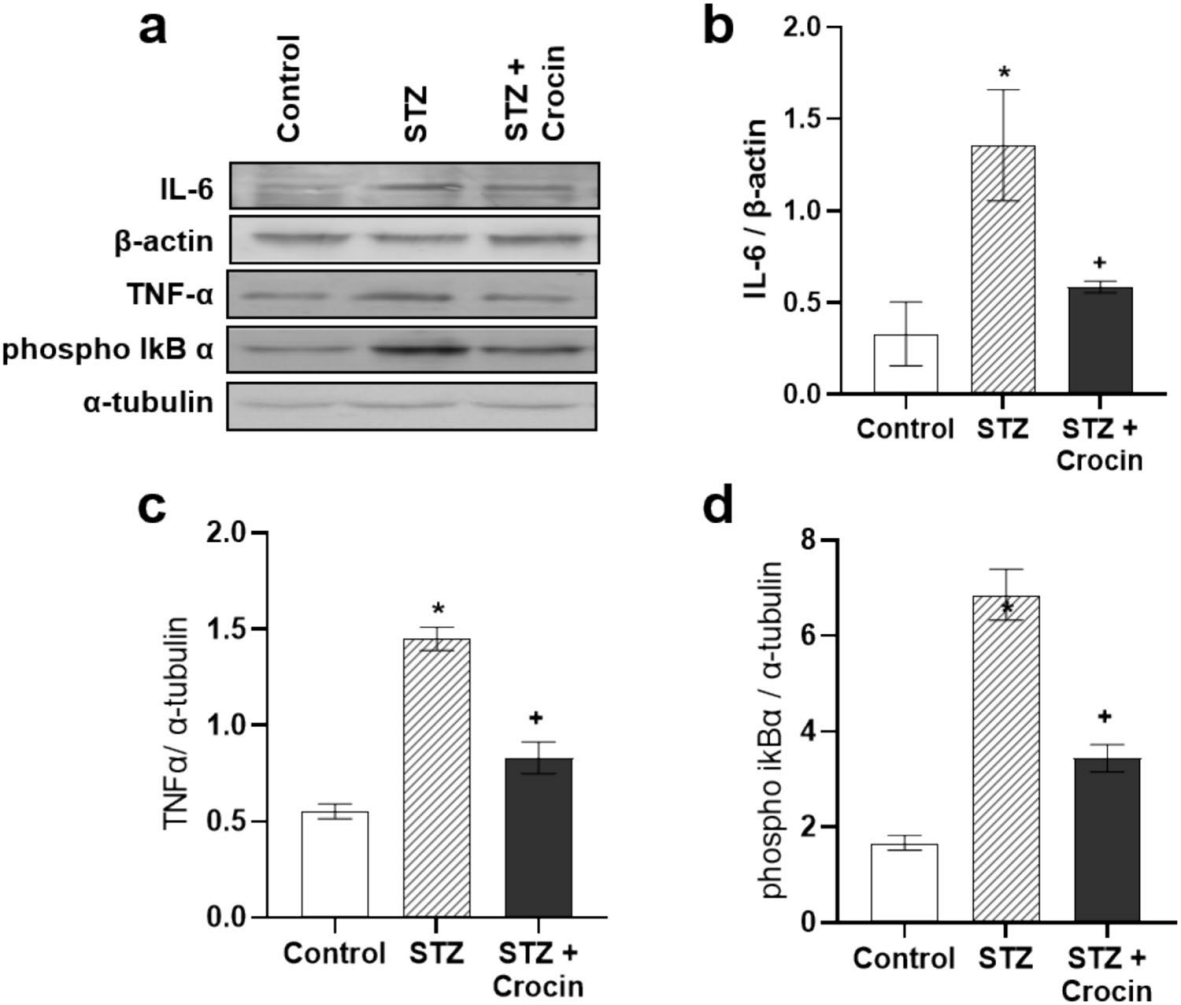
Treatment with crocin alleviates myocardial inflammation markers. Representative western blot images (**a**) and densitometric analysis of (**b**) IL-6 (normalized to β-actin), **c** TNFα (normalized to α-tubulin) and **d** phosphorylated IkBα (normalized to α-tubulin), in the hearts of control, STZ - treated and STZ + crocin-treated rats. Data are presented as mean ± SEM; *p < 0.05 compared with the control group, +*p* < 0.05 compared with the STZ-treated group; one-way ANOVA followed by Student-Newman-Keuls multiple comparisons test; *n* = 4

The metabolic imbalance in the diabetic myocardium is tightly associated with severe oxidative stress and intense inflammation, both of which further impair cardiac function. To evaluate the effect of crocin on cardiac inflammatory markers, we determined protein levels of two proinflammatory cytokines, IL-6 and TNFα, as well as phosphorylation levels of IkBα, which were increased by approximately threefold in the diabetic hearts (Figure 4a–d). When phosphorylated, IkBα releases NF-κB enabling NF-κB-mediated transcriptional activation of target genes involved in inflammatory responses [29]. However, crocin treatment significantly reduced IL-6 (Figure 4b) and TNFα (Figure 4c) protein levels, as well as IkBα phosphorylation (Figure 4d). Taken together, these findings support the anti-inflammatory role of crocin in the diabetic heart.

## Discussion

The findings of the present study demonstrate that crocin alleviates diabetes-induced metabolic disturbances by normalizing the balance between glucose and fatty acid metabolism and attenuating inflammation in the hearts of STZ-induced diabetic rats. Although previous studies have highlighted the beneficial role of crocin in systemic metabolic disorder in diabetes [16, 17, 30], this is the first study to demonstrate its effect on myocardial energy metabolism, thereby revealing a novel cardioprotective role. Given that fluctuations in intracellular glucose and lipid levels trigger cardiomyocyte apoptosis under diabetic conditions, the beneficial effects of crocin on cardiac metabolism observed in this study may explain its previously described anti-apoptotic properties in the heart [19].

In both insulin-deficient and insulin-resistant DM, myocardial energy metabolism is impaired, contributing to contractile dysfunction, diabetic cardiomyopathy, and ultimately heart failure. Metabolic perturbations in the diabetic heart include reduced glucose uptake and utilization, coupled with excessive fatty acid (FA) uptake and oxidation, leading to lipid accumulation and severe lipotoxicity [5, 31, 32]. Consistent with the increased reliance on FAs as a cardiac energy source, an upregulation of peroxisome proliferator-activated receptors (PPARα and PPARδ), the major transcriptional regulators of myocardial lipid homeostasis [28, 32, 33], was observed in diabetic hearts. The upregulation of these nuclear receptors was also accompanied by an upregulation of CD36, a PPAR-regulated membrane transporter responsible for intracellular FA uptake. Upon treatment with crocin, the significant decrease in PPAR protein levels reflects a reduction in FA availability in the diabetic heart, while the downregulation of CD36 suggests a reduction in lipid uptake closer to basal control levels. Moreover, crocin administration decreased FA oxidation (FAO) rates, as evidenced by reduced 3-hydroxyacyl-CoA dehydrogenase (HOAD) activity, ultimately leading to a reduction in intramyocardial triglyceride content. These effects protect the diabetic heart from lipotoxicity, which is one of the major contributing factors to diabetic cardiomyopathy. As has been previously demonstrated in genetically modified animal models, lipid accumulation in the heart impairs cardiac contractility even in the absence of systemic metabolic imbalances [34]. Furthermore, excessive reliance on FAs in the diabetic heart is known to reduce cardiac efficiency and contractility [35]. Our current data suggests a previously unrecognized protective role of crocin against cardiac lipid accumulation through normalization of FA uptake, oxidation and storage, the derangement of which is related to severe lipotoxicity including oxidative stress and inflammation. Therefore, these findings could implicate crocin for further research in patients with lipotoxicity-associated cardiomyopathies.

DM is associated with decreased myocardial Akt phosphorylation and activation and impaired GLUT-4-mediated glucose uptake. Under normal conditions, activated Akt promotes GLUT4 membrane translocation, thereby facilitating glucose uptake in cardiomyocytes [36–38]. In addition to its role in glucose metabolism, activation of PI3K/Akt signaling mitigates apoptosis by upregulating Bcl-2, an anti-apoptotic effector, thereby preventing cardiomyocyte death [39]. Crocin administration strongly enhanced both Akt phosphorylation and GLUT-4 membrane translocation in the diabetic myocardium and cardiac myocytes. The stimulation of glucose metabolism toward basal levels is further evidenced by the normalization of pyruvate kinase (PK) activity in crocin-treated diabetic hearts. Essential fine-tuning between glucose and lipid metabolism is regulated by pyruvate dehydrogenase (PDH) and its inhibitor, pyruvate dehydrogenase kinase 4 (PDK4). Notably, crocin downregulated PDK4 expression, which alleviates PDH inhibition, thereby promoting glycolytic flux into the tricarboxylic acid (TCA) cycle. This metabolic shift favors glucose oxidation over FA β-oxidation, restoring a balance resembling that of non-diabetic hearts. These findings suggest that crocin effectively rescues impaired myocardial energy metabolism and restores equilibrium between glucose and lipid utilization.

Chronic inflammation resulting from hyperglycemia, oxidative stress, and metabolic dysfunction is a hallmark of the diabetic heart. Pro-inflammatory cytokines such as TNF-α and IL-6 are typically secreted by cardiomyocytes in response to pathological stimuli [40] under the control of nuclear factor-kappa B (NF-κB) [41]. Under physiological conditions, NF-κB remains bound to its inhibitor, IκB, but upon phosphorylation, IκB dissociates, allowing NF-κB to translocate to the nucleus and activate inflammatory target genes [42]. Our results demonstrate that crocin treatment significantly reduced STZ-induced elevations in TNF-α, IL-6, and phosphorylated IκB levels. Additional studies further support crocin’s anti-inflammatory effects in various animal models [14]. Furthermore, intraperitoneal administration of crocin reduced TNF-α, IL-6, and IL-1β levels in the renal tissues and serum of aged rats [43], while crocetin, a crocin metabolite, has been shown to attenuate ischemia/reperfusion injury and decrease TNF-α, and IL-10 levels in rat hearts [44]. Our data suggest that crocin exerts an anti-inflammatory effect in the diabetic heart, potentially mediated by its role in restoring metabolic balance.

## Conclusions

**Fig. 5:**
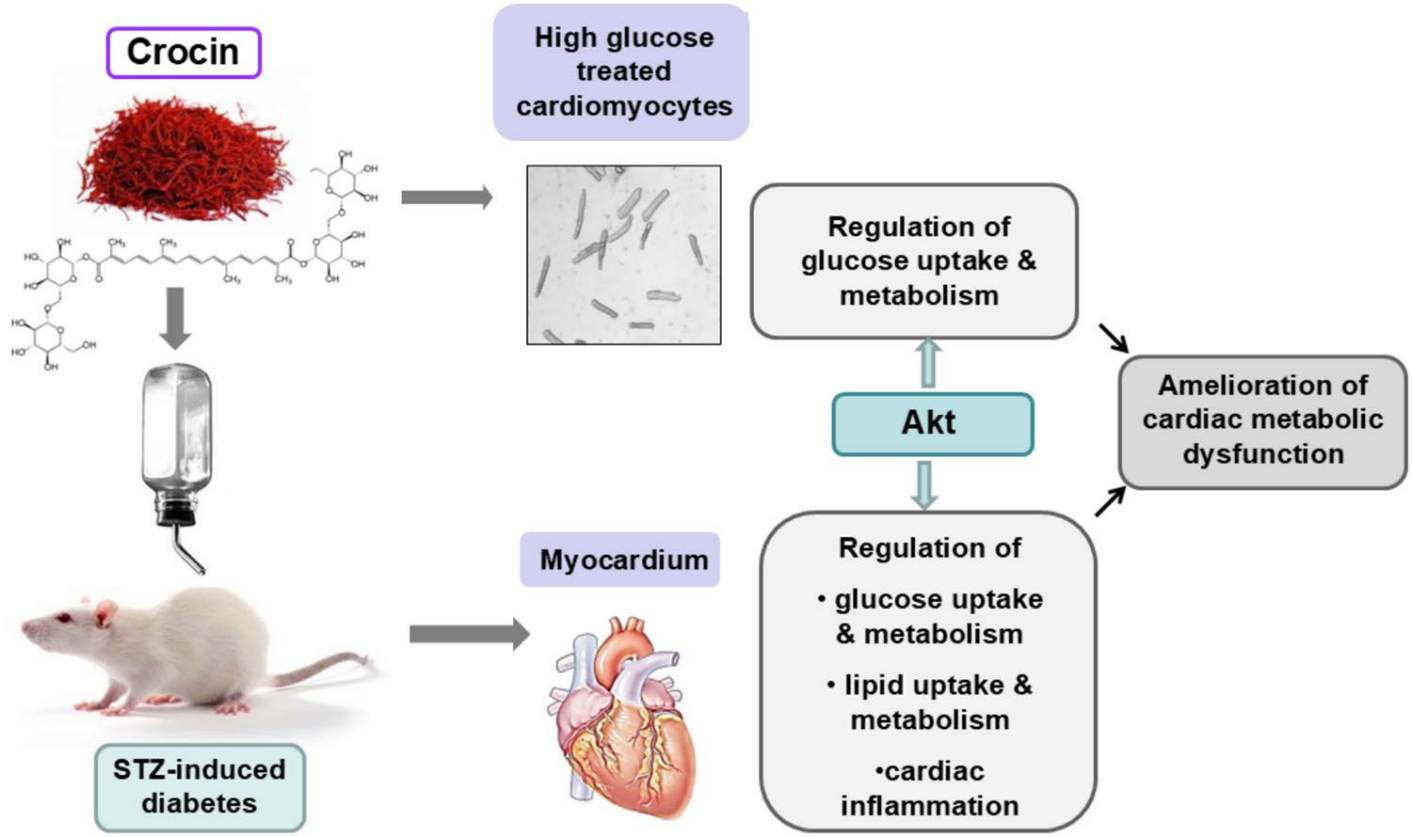
Proposed role of crocin in modulating the cardiac metabolic profile in the STZ-induced diabetic rat model. Crocin promotes a balanced utilization of glucose and fatty acids in the diabetic heart by stimulating Akt activation, thereby restoring metabolic homeostasis

In conclusion, our results demonstrate that crocin treatment restores myocardial metabolic balance potentially by activating Akt signaling. While the precise molecular mechanisms underlying crocin’s metabolic benefits remain to be fully elucidated, the observed increase in Akt phosphorylation/activation in diabetic myocardium and cardiomyocytes suggests that Akt may serve as a critical mediator of crocin-induced metabolic improvements (Fig. 5). Given the pivotal role of metabolic homeostasis in diabetes progression, oral crocin administration offers promising evidence as a natural therapeutic supplement. By further elucidating crocin’s metabolic mechanisms, this research paves the way for the development of natural compounds as adjunct therapies for diabetic cardiomyopathy.

## Limitations of the study

The study demonstrates crocin’s ameliorative effects on diabetic complications in rats, 6 weeks after STZ administration. However, since diabetes is a progressive and highly heterogeneous metabolic disorder, our findings should be interpreted with caution when applied to different stages of acute or chronic diabetes. Moreover, since only male rats were included in this study, further investigation is needed to validate the effects of crocin treatment in females. Although oral crocin administration may influence non-cardiac tissues, these effects were not evaluated in the present study and warrant further investigation, including their potential implications for heart function.

## Data Availability

All data supporting the findings of this study are available within the paper.

## Abbreviations

CD36: Cluster of differentiation 36
CS: Citrate synthase
DEPC: Diethylpyrocarbamate
DM: Diabetes mellitus
DTT: Dithiothreitol
E64: Trans-epoxy succinyl-L-leucyl-amido-(4-guanidino) butane
FA: Fatty acids
FAO: Fatty acid oxidation
GLUT-4: Glucose transporter type 4
HEPES: 4-(2-Hydroxyethyl)-1-piperazineethanesulfonic acid
HOAD: 3-Hydroxyacyl CoA dehydrogenase
HDL: High density lipoprotein
HG: High glucose
LDL: Low density lipoprotein
LDH: Lactate dehydrogenase
PDH: Pyruvate dehydrogenase
PDK4: Pyruvate dehydrogenase kinase
PK: Pyruvate kinase
PMSF: Phenyl methyl sulfonyl fluoride
PPAR: Peroxisome proliferator-activated receptor
SDS: Sodium dodecyl sulfate
STZ: Streptozotocin
TNF-α: Tumor necrosis factor *α*
IL-6: Interleukin 6
